# Editorial: Molecular informatics in personalized medicine, volume II

**DOI:** 10.3389/fmed.2026.1861955

**Published:** 2026-05-28

**Authors:** J. Francis Borgio, Hari S. Sharma, Noor B. Almandil, Sayed AbdulAzeez, Peter J. van der Spek

**Affiliations:** 1 Department of Genetic Research, Institute for Research and Medical Consultations (IRMC), Imam Abdulrahman Bin Faisal University, Dammam, Saudi Arabia; 2 Clinical Bioinformatics, Department of Pathology, Erasmus Medical Center, University Medical Center, Rotterdam, Netherlands; 3 Department of Clinical Pharmacy Research, Institute for Research and Medical Consultations (IRMC), Imam Abdulrahman Bin Faisal University, Dammam, Saudi Arabia

**Keywords:** immunoinformatics analysis, metabolomics, metagenomics, microRNA, precision medicine and genomics, whole genome sequencing (WGS)

## 1 Introduction

The second volume of our Research Topic, “*Molecular informatics in personalised medicine*,” comes out at a key point in the development and management of healthcare. We are observing a shift from reactive, uniform clinical models to a proactive, data-driven framework wherein the molecular fingerprint of a patient determines respective therapeutic strategy. This volume has five important papers that show how high-throughput data, like genomic long-reads, metabolomic profiles, immunoinformatics, and metagenomics are being used to solve traditional challenges with diagnosis and treatment. Personalized medicine is no longer a dream for the future; it is now a clinical reality based on molecular informatics. The researchers in this issue of the journal show that we can now achieve levels of precision seen never before in managing complicated diseases like prostate cancer, cardiovascular disease, metabolic syndromes as well as infectious diseases by combining different types of biological data with advanced computational analysis and machine learning. It is hoped that by integrating multi-omics with artificial intelligence would serve in establishing precision therapeutic strategies those will be not only be a mile stone in the cure but also serve as establishing policies.

## 2 Contributions

### 2.1 Real-time precision with nanopore sequencing

A recurring theme in this volume is the demand for more accessible and rapid genomic tools. Massaiu et al. discuss a significant bottleneck in genetic testing. The constraints of conventional short-read sequencing in elucidating complex genomic regions. Their research focuses on the PCSK9 gene, a principal regulator of LDL cholesterol and a key target for cardiovascular risk mitigation. The team created a workflow for fast, long-read targeted sequencing of a ~25 kb locus by using Oxford Nanopore Technologies (ONT). This method is revolutionary because it allows you look at ultra-long DNA fragments in real time, bypassing the need for complex assembly required by short-read methods. The study is important because it gives a bioinformatic framework for clinical use and compares hardware like the Flongle and MinION flow cells to high-accuracy basecalling models. Their discovery that a Super High Accuracy (SUP) model, when used with the Longshot variant caller, gets a 100% F1-score for single nucleotide variations (SNVs) sets a new standard for diagnostic accuracy. This study indicates that the future of genomic diagnostics is in long reads, opening the way for cost-effective, high-throughput diagnostic panels that may expand from cardiology to oncology and rare disease screening.

### 2.2 Clarifying phenotypic uncertainty using metabolomics

Genomics provides the blueprint, while metabolomics gives a snapshot of how the body works in real time. Al Mahdi et al. illustrate this by addressing the diagnostic challenge of hypercholesterolemia within the Saudi population. Familial hypercholesterolemia (FH) and non-genetic hypercholesterolemia (HC) are clinically indistinguishable, both characterized by perilously elevated LDL-C levels. But the molecular mechanisms behind them are very different, so they need different ways to be managed. Utilizing high-resolution untargeted metabolomics (UPLC-Q-TOF/MS), the researchers identified 1,359 metabolites and demonstrated that FH is distinctly characterized by dysregulated steroidogenesis and bile acid biosynthesis. They suggested that 17α-hydroxyprogesterone (17α-OHP) and cholic acid could be useful biomarkers for telling the difference between genetic and non-genetic cases. This distinction has significant clinical implications: whereas hypercholesterolemia can be managed through lifestyle-oriented interventions targeting oleic and linoleic acid metabolism, familial hypercholesterolemia patients may necessitate targeted therapies, such as advanced bile acid sequestrants, to resolve their particular metabolic challenges.

### 2.3 Immunoinformatics for designing the next generation of vaccines

Informatics-driven design is also changing the field of oncology. Runtunuwu et al. propose a multi-faceted computational strategy to create a therapeutic vaccine for prostate cancer, a malignancy characterized by tumor heterogeneity and immune evasion. The authors developed a multi-epitope construct aimed at three principal biomarkers such as PSMA, STEAP1 and B7H3, as opposed to single-antigen vaccines. The strictness of their immunoinformatics pipeline, which includes epitope prediction, population coverage analysis and molecular dynamics, made sure that the vaccine is safe, non-allergenic, with a predicted HLA allele coverage of 97.51%. Molecular docking simulations showed that this peptide-based vaccine has strong binding affinities to important immune receptors. This suggests that it could be a cost-effective and scalable alternative to autologous cell therapies like Sipuleucel-T. This research represents a preliminary advancement toward personalized cancer immunotherapy, wherein vaccines are customized to target the most common and stable antigens present within a particular tumor microenvironment.

### 2.4 Metagenomics for navigating the diagnostic fog

Personalized medicine is also important for saving lives in emergency rooms. Chen et al. present a complicated case of a patient with Systemic Lupus Erythematosus (SLE) who experienced life-threatening complications. The clinical presentation was complicated by the concurrent symptoms of an autoimmune flare and hemophagocytic syndrome (HPS), making the identification of underlying infections nearly unfeasible via conventional serology. The breakthrough occurred via metagenomic Next-Generation Sequencing (mNGS) of both blood and cerebrospinal fluid. This untargeted method found high levels of *Toxoplasma gondii* DNA, which showed that the infection was spreading and would have stayed hidden otherwise. Rapid molecular identification enabled the prompt optimisation of antimicrobial therapy and the secure reduction of immunosuppressants, ultimately resulting in a favorable outcome. This case illustrates the essential role of mNGS in the management of immunocompromised patients, where deciphering host-pathogen interactions in clinical settings is vital for survival.

### 2.5 microRNA signatures for early identification of metabolic transition

Finally, Atamni et al. examine the regulatory framework of Type 2 Diabetes (T2D) via circulating microRNAs (miRNAs). Their research identified 141 miRNAs that were significantly dysregulated in pre-diabetic individuals, suggesting that molecular alterations transpire prior to the emergence of clinical symptoms. A distinctive aspect of this research is the focus on sex-stratified analysis. The finding that males and females exhibit distinct miRNA expression patterns during glycemic dysregulation underscores a vital principle of personalized medicine: biological sex is a critical covariate that must be integrated into biomarker development. By associating these miRNA signatures with clinical variables such as vitamin D levels and platelet activity, the authors present a comprehensive perspective of the diabetic trajectory, revealing novel avenues for early intervention and prevention.

## 3 Conclusion

All the articles embedded in this volume together show that molecular informatics is what makes personalized medicine appropriate. The use of computational tools in clinical practice is now a reality in saving lives, whether it's by clearing up the diagnostic fog of rare infections with mNGS or by creating global cancer vaccines with immunoinformatics. As we look ahead, there could still be a lot of problems to solve. We need to standardize bioinformatics protocols, ensure that these technologies are cost-effective for global health and keep testing *in silico* predictions with strict clinical trials. But the basic work shown here provides a clear path forward. It is no longer a matter of intuition what the future of medicine will be; it is now a matter of information that is accurately captured, expertly analyzed and eventually personally applied.

